# Rational Engineering and Preclinical Evaluation of Neddylation and SUMOylation Site Modified Adeno-Associated Virus Vectors in Murine Models of Hemophilia B and Leber Congenital Amaurosis

**DOI:** 10.1089/hum.2019.164

**Published:** 2019-11-26

**Authors:** Shubham Maurya, Bertin Mary, Giridhara R. Jayandharan

**Affiliations:** Department of Biological Sciences and Bioengineering, Indian Institute of Technology, Kanpur, Uttar Pradesh, India.

**Keywords:** rational engineering, AAV, capsid, Neddylation, SUMOylation, hemophilia, retinal degeneration

## Abstract

Synthetic engineering of viral vectors such as adeno-associated virus (AAV) is crucial to overcome host transduction barriers observed during clinical gene therapy. We reasoned that exploring the role of cellular ubiquitin-like modifiers (UBLs) such as Neddylation or SUMOylation during AAV transduction could be beneficial. Using a combination of *in silico* biochemical and molecular engineering strategies, we have studied the impact of these UBLs during AAV2 infection and further developed Neddylation or SUMOylation site–modified AAV vectors and validated them in multiple disease models *in vitro* and *in vivo*. Hepatic gene transfer of two novel vectors developed, K105Q (SUMOylation-site mutant) and K665Q (Neddylation-site mutant), demonstrated a significantly improved human coagulation factor (F) IX expression (up to two-fold) in a murine model of hemophilia B. Furthermore, subretinal gene transfer of AAV2-K105Q vector expressing *RPE65* gene demonstrated visual correction in a murine model of a retinal degenerative disease (rd12 mice). These vectors did not have any adverse immunogenic events *in vivo*. Taken together, we demonstrate that gene delivery vectors specifically engineered at UBLs can improve the therapeutic outcome during AAV-mediated ocular or hepatic gene therapy.

## Introduction

Gene therapy using viral vectors has recently emerged as a potent tool in the field of molecular medicine. Among the gene delivery systems available, recombinant adeno-associated viruses (AAV) are attractive due to their relatively nonpathogenic nature, ability to transduce dividing and nondividing cells, as well as their long-term expression in infected cells.^1^ The availability of multiple AAV serotypes (1–10) to target a variety of tissues in humans also greatly improves the versatility of this delivery system.^2^ AAV2 is the prototype vector that was first described,^3^ and ∼6% of clinical gene therapy trials have been conducted with this vector.^4^ AAV2 serotype has been used for gene therapy of hemophilia B^5^ and more recently successfully in patients with Leber congenital amaurosis (LCA type 2).^6^

In the hemophilia B trial, the CD8^+^ T cell-mediated immune response in patients who received high doses of AAV2 vectors^5^ has now precluded the use of AAV2 for hepatic gene transfer. Subsequently, other serotypes such as AAV8 have demonstrated substantial clinical benefits in patients with hemophilia B.^7^ However, clinical observations in both the hepatic^5^ and ocular gene therapy trials with AAV2^8^ have revealed that low doses of AAV2-FIX vectors (up to 4 × 10^11^ vector genomes [vgs]/kg) are immunologically safe but therapeutically suboptimal; while in LCA2 patients who received a low dose (1 × 10^11^ vgs/eye) of AAV2-RPE65 vectors, there was a negligible vision rescue. Taken together, these data suggest that strategies to improve the transduction efficiency of AAV2 vectors are needed, so that they are therapeutic at low vector doses. To achieve this, it is crucial to study the biology of AAV2-host interactions so that optimal vectors could be synthesized and tested.

AAV2 is a nonenveloped virus containing a single-stranded genome of ∼4.7 kb in size. AAV2 belongs to the Parvoviridae family and genus *Dependovirus*.^9^ The capsid of AAV2 has an icosahedral symmetry, which consists of VP1, VP2, and VP3 proteins in a ratio of 1:1:10 generated from alternative splicing of AAV genome and assembly activating protein.^10–12^ AAV2 infects the host cell by binding to cell surface receptors and undergoes clathrin-mediated endocytosis followed by its intracellular trafficking and nuclear entry.^13^ It is well recognized that ∼30% of AAV2 vectors can successfully enter the nucleus while the rest are lost to intracytoplasmic degradation mechanisms.^14^ Post-translational modifications (PTMs) like phosphorylation and ubiquitination are known to contribute to vector loss by initiating proteasome degradation pathways.^15^ AAV2 vectors mutated at potential phosphorylation and ubiquitination sites have been developed to circumvent these PTMs, which led to a substantial increase in their transduction *in vitro* and *in vivo*.^16^ However, the presence and potential of other PTMs such as ubiquitin-like modifiers (UBLs) including Neddylation and SUMOylation on AAV2 capsid and their possible implication on AAV2 transduction is largely unexplored.

The process of Neddylation is a reversible mechanism where NEDD8 (neural precursor cell expressed developmentally downregulated-8) protein binds to its substrate and alters the function of its targets. Neddylation is operated by a set of conserved enzymes named E1, E2, and E3 ligases. In the first step, NEDD8 is activated through an ATP-dependent mechanism by an E1 enzyme (APPBP1/UBA3).^17^ Furthermore, NEDD8 is transferred to an E2 conjugating enzyme (UBC12),^18^ and finally, E3 ligase transfers NEDD8 to its substrate.^19^ Neddylation majorly targets cullin-RING ligase (CRLs) as its substrate, which results in their activation. CRLs belong to the family of E3 enzymes of ubiquitin pathway,^20^ thus Neddylation independently or together activate ubiquitination. Similarly, SUMO (small ubiquitin-like modifier) is an 11 kDa protein, which shares structural similarity to ubiquitin protein.^21^ The process of SUMOylation involves a enzymatic-cascade and involves maturation, activation, conjugation, and ligation of SUMO proteins to its substrate.^21^ SUMO proteins mature by C-terminal cleavage mediated by a family of SENP (sentrin/SUMO-specific protease) enzymes. It undergoes an ATP-dependent activation by E1 (SAE1/SEA2) activating enzyme^22^ followed by binding to E2 conjugating enzyme (UBC9) via a thio-ester linkage.^23^ Finally, it binds to its substrate at consensus ψKXE (where ψ represents a hydrophobic amino acid, and X represents any amino acid) lysine residue with the help of E3 ligase.^24,25^ However, it must be noted that SUMOylation is known to occur at this consensus sequence in only ∼75% of targets, whereas ∼25% of SUMOylation modifications can occur in nonconsensus sites.^26^

While the role of Neddylation and SUMOylation in modulating the life cycle of several viruses such as human papillomavirus, Herpes simplex virus type-1 (HSV-1), and influenza A virus^27,28^ are known, only one study based on SUMOylation is available in the context of AAV.^29^ A recent study employed siRNA-mediated knockdown of *SAE2* and *UBC9* targets in the SUMOylation pathway and demonstrated an increase in the transduction of AAV2 in an *in vitro* model of HeLa cells.^29^ Given the paucity of data on UBLs modulating AAV infection, we reasoned that exploring the role of Neddylation or SUMOylation during AAV transduction would be rewarding to augment its gene transfer efficiency. Thus, using a series of molecular and biochemical strategies, we have deciphered the role of these UBLs during AAV2 infection and synthesized improved gene delivery vectors that demonstrate therapeutic efficiency during hepatic and ocular gene therapy.

## Materials and Methods

### Cell lines, reagents, and animal models

Human hepatocellular carcinoma (Huh7) cell line was a kind gift from Dr. Saumitra Das, IISc, Bangalore. Human cervical carcinoma cells (HeLa) were obtained from American Type Culture Collection (ATCC, Manassas, VA). Adult retinal pigmental epithelium (ARPE)19 cell line was a kind gift from Dr. Sowmya Parameswaran and Dr Krishnakumar, Sankara Nethralaya, Chennai. The cells were cultured in complete Iscove's modified Dulbecco's medium (IMDM; Gibco, Life Technologies, Carlsbad, CA) with 10% fetal bovine serum (Gibco) at 37°C with 5% CO_2_, supplemented with 10 μg/mL each of ciprofloxacin (HiMedia Laboratories, Mumbai, India) and piperacillin (MP Biomedicals, Santa Ana, CA). Small-molecule inhibitors for NAE1 protein (MLN4924) were purchased from Calbiochem (Merck, Kenilworth, NJ). Intravenous immunoglobulin was procured from Baxter Biosciences (Deerfield, IL). SYBR green qPCR master mix was purchased from Promega (Madison, WI). C57BL6/J, rd12, and hemophilia B mice (B6.129P2-*F9^tm1Dws^*/J) were procured from Jackson Laboratory (Bar Harbor, ME). All animal experiments were approved by the IIT-Kanpur Institutional Animal Ethics Committee. The animal experiments were carried out in accordance with the relevant guidelines and regulations.

### Drug assays *in vitro*

Approximately 3 × 10^4^ HeLa cells were seeded in a 24-well plate and incubated for 12 h. Cells were either mock-treated or treated with a Neddylation inhibitor, MLN4924 (Calbiochem; Merck), at a concentration of 4.7 nM, 470 nM, and 1 μM and incubated at 37°C for 3 h. After incubation, cells were infected with self-complementary (sc) AAV2-EGFP (enhanced green fluorescent protein) vectors at a multiplicity of infection (MOI) of 5 × 10^3^ vgs/cell for 3 h in IMDM at 37°C. Forty-eight hours later, the transgene (GFP) expression was quantified by flow cytometry (BD Accuri C6 Plus; BD Biosciences, Franklin Lakes, NJ).

### Targeted transcriptome analysis for Neddylation and SUMOylation pathways

Total RNA from each of the treated condition in HeLa cells was extracted by TRIzol reagent (Thermo Fisher, Waltham, MA). About 1 μg of RNA was used to generate cDNA by using the Verso cDNA Synthesis Kit (Thermo Fisher). The primers used for Neddylation and SUMOylation gene targets were procured from Imperial Life Sciences (ILS, Gurgaon, India). Transcript levels of Neddylation target genes *APPBP1*, *UBA3*, *UBC12*, *NEDD8* (Supplementary Table S1) and SUMOylation target genes SAE1, SAE2, UBC9, SUMO1 (Supplementary Table S2) were measured by quantitative polymerase chain reaction (qPCR) in a CFX96 Real-Time System (Bio-Rad, Hercules, CA) with β-actin as an endogenous control for the normalization of data.

### *In silico* prediction of Neddylation and SUMOylation sites on AAV2 capsid

AAV2 VP1 capsid protein sequence (Protein ID: YP_680426.1) was used to predict Neddylation and SUMOylation targets. Neddylation sites were predicted with online tool NeddyPreddy (http://NeddyPreddy.sabanciuniv.edu).^30^ This tool has medium and high threshold levels based on output generated by the support vector machine.^30^ For our analysis, we set the threshold to a medium setting to capture relatively high confidence targets. Online tools GPS-SUMO^31,32^ and SUMOplot (www.abgent.com/sumoplot) was used to predict SUMOylation sites.

### AAV vector production

The top five sites predicted for Neddylation (score >0.3) and SUMOylation (score >0.5) by NeddyPreddy and both GPS-SUMO and SUMOplot were chosen for further site-directed mutagenesis (Supplementary Tables S3–S5). Neddylation and SUMOylation targets were mutated from lysine to glutamine (K>Q) residues by using QuikChange II XL Site-Directed Mutagenesis Kit (Agilent Technologies, Santa Clara, CA) as per the manufacturer's instructions using primers detailed in Supplementary Tables S6 and S7. Viral vectors were packaged and purified as described earlier.^33^ Briefly, forty 150-mm^2^ dishes, 80% confluent with AAV-293 cells, were transfected with AAV2 (rep/cap) or AAV2 mutant capsid vectors, transgene vectors containing enhanced green fluorescent protein (EGFP) or human factor IX or retinal pigment epithelium 65 (p.dsAAV2 CBa EGFP or p.dsAAV2 LP1 h.FIX, a kind gift from Dr. Amit Nathwani, UCL or p.ssAAV2 CMV RPE65, a kind gift from Dr. J. Bennett, UPenn) and AAV-helper (p.helper) vectors. Cells were collected 68–72 h post-transfection, lysed, and treated with Benzonase Nuclease (25 units/mL; Sigma–Aldrich, St. Louis, MI). Furthermore, the vectors were purified by iodixanol gradient ultracentrifugation (OptiPrep; Sigma–Aldrich) followed by column chromatography (HiTrap SP column; GE Healthcare Life Sciences, Chicago, IL). The vectors were concentrated to a final volume of 0.5 mL in phosphate-buffered saline (PBS) using Amicon Ultra 10K centrifugal filters (Millipore, Burlington, MA). Vectors were then quantified by qPCR as described earlier.^34^

### Transduction assays

About 3 × 10^4^ cells of Huh7, ARPE19, and HeLa cells were mock-infected or infected with scAAV2-EGFP and scAAV2-EGFP mutant vectors at an MOI of 5 × 10^3^ vgs/cell for 3 h. Two days later, the transgene (GFP) expression was quantified by flow cytometry (CyFlow, Sysmex-Partec, Kobe, HP, Japan, or BD Accuri C6 Plus; BD Biosciences).

### Virus entry assay

HeLa cells were seeded at a density of 1 × 10^5^ cells/well in a 24-well plate. Cells were then mock infected or infected with scAAV2-EGFP or AAV2 mutant viruses at an MOI of 1 × 10^4^ vgs/cell. Three hours later, infected cells were collected by trypsinization, and genomic DNA was isolated by ethanol precipitation. Viral genomes were quantified against appropriate plasmid standards and with “PolyA site” as a target in vector backbone for amplification by qPCR.^34^

### Western blot analysis

About 1.42 × 10^10^ vgs of AAV vectors were loaded onto a denaturing SDS-PAGE (sodium dodecyl sulfate–polyacrylamide gel electrophoresis) gel. Resolved proteins were further transferred into polyvinylidene fluoride (PVDF) membrane (Pall Corporation, Port Washington, NY). Subsequently, the membrane was blocked with 5% bovine serum albumin (BSA) for 1 h. Membranes were then probed with anti-AAV (B1) (1:500; Fitzgerald, North Acton, MA) or anti-SUMO-1 (1:1,000; Sigma–Aldrich) primary antibodies and detected with an anti-mouse horseradish peroxidase (HRP)-conjugated secondary antibody (1:2,500; Abcam, Milton, Cambridge, United Kingdom). The signals were developed by chemiluminescent substrate (SuperSignal™ West Pico PLUS; Thermo Scientific). Densitometric quantification was performed by using ImageJ^35^ in three different blots with two measurements at least for each blot developed.

### Dot blot analysis

For Neddylation detection, 1.42 × 10^10^ vgs of AAV2-WT and AAV2-K665Q were spotted in the PVDF membrane in equal volume. For loading control, vectors were preincubated with 0.4 N NaOH at room temperature for 30 min. Membranes were blocked in 5% BSA for 30 min followed by incubation in primary antibody, anti-NEDD8 (1:500; Santa Cruz Biotechnology, Dallas, TX) and anti-AAV B1 (1:500; Fitzgerald), for 1 h. Subsequently, membranes were washed and incubated with HRP-conjugated secondary antibody (1:2,500; Abcam) for 30 min. After adequate washing, the blots were probed with a chemiluminescence detection kit (Thermo Scientific). Dot blots were performed twice, and representative images are shown. Densitometric analysis was performed using the ImageJ software with three different exposures and normalized to loading (B1) control.

### Hepatic gene transfer in hemophilia B mice

About 5 × 10^10^ vgs of scAAV2 vectors or scAAV2 mutant vectors containing LP1 promoter-driven human *FIX* (scAAV2 LP1-h.FIX) were administered into 6- to 8-week-old hemophilia B mice, via the tail vein. PBS was administered into the control group of hemophilia B mice. Five and 8 weeks after gene transfer, retro-orbital blood collection from all animals was performed and plasma isolated by standard methods. To assay the h.FIX activity in murine plasma (*n* = 5 animals per group), we performed an enzyme-linked immunosorbent assay (ELISA) using a commercial kit (Asserachrom IX: Ag; Diagnostica Stago, Asnières, France).

### Immunohistochemistry

For immunostaining of human FIX, murine liver samples were harvested after 9 weeks of hepatic gene transfer. Samples were embedded in OCT media (Polyfreeze; Sigma–Aldrich), sectioned at 10 μm thickness, and fixed in 4% paraformaldehyde for 15 min at room temperature. Slides were washed with PBS and blocked in a solution containing 10% normal donkey serum (Santa Cruz Biotechnology), 0.2% Triton X-100 (Sigma–Aldrich) diluted in PBS for 1 h at room temperature. Subsequently, sections were incubated with goat anti-human FIX antibody (1:100; Affinity Biologicals, Hamilton, ON, Canada) overnight at 4°C. After washing thrice, the slides were incubated with donkey anti-goat Cy3 antibody (Jackson ImmunoResearch, West Grove, PA) at dilution of 1:500 for 1.5 h at room temperature. Sections were washed thrice and mounted with Fluoroshield™ with DAPI (Sigma–Aldrich). Images were acquired by Leica DMi8 confocal microscope (Wetzlar, Germany).

### Immune assays

To examine the immunogenicity associated with hepatic gene transfer of AAV2 vectors, we enumerated the T cell, B cell, and T-reg cells in hemophilia B mice that received gene therapy (*n* = 5 per group). Briefly, peripheral blood from hemophilia B mice was collected 9 weeks after gene transfer. After RBC lysis (155 mM NH_4_Cl, 12 mM NaHCO_3_, and 0.1 mM EDTA), samples were incubated with a combination of FITC-labeled anti-CD3, PE-labeled anti-CD8, PerCP-labeled anti-CD4, and APC-labeled anti-CD19 (BD Biosciences) antibodies for 30 min at room temperature, and percentage CD3^+^, CD4^+^, CD8^+^, and CD19^+^ cells were assessed by flow cytometry (BD Accuri C6 Plus). To profile the T-reg cells in murine splenocytes, ∼2 million cells were stained with PerCP-labeled anti-CD4 and APC-labeled anti-CD25 and PE-conjugated Foxp3 antibodies as per the manufacturer's protocol (BD Biosciences).

### ELISPOT assay

Splenocytes from control (*n* = 10), AAV2 (*n* = 10) or mutant vector treated (*n* = 5) mice were harvested at 9 to 12 weeks after hepatic gene transfer and samples processed as described earlier.^36^ Briefly, after RBC lysis, ∼1 × 10^6^ viable splenocytes were stimulated with 2 μg/mL of AAV2 capsid T cell epitope-specific peptide (SNYNKSVNV; JPT Peptide Technologies, GmbH, Germany) and seeded into IFN-γ antibody–precoated ELISPOT plate (MabTech, Cincinnati, OH). Concanavalin A (2 μg/mL) was used as positive control for the assay. After 36 h of incubation at 37°C, spots were developed using BCIP/NBT. Spot-forming units and the images were captured in an ELISPOT reader (AID Reader, GmbH, Germany).

### Ocular gene transfer and fluorescence imaging

Eyes of C57BL6/J mice were dilated using phenylephrine and tropicamide (Sunways India Pvt. Ltd., Mumbai, India). Mice were anesthetized by intraperitoneal injection of ketamine (80 mg/kg) and xylazine (12 mg/kg). For intravitreal administration, an opening was created at sclera near limbus by an insulin syringe, and 1–2 μL of vectors was injected through the same opening by Hamilton syringe fitted with 33-gauge beveled needle. After injections were completed, tobramycin (Sunways India Pvt. Ltd.) was applied to the eyes. Fluorescence imaging was performed after 4 weeks of vector administration (*n* = 4-7 eyes per group) in a Micron IV imaging system as per manufacturer's instructions (Phoenix Research Lab, Pleasanton, CA). Intensity was set at maximum and gain was set at 15 db; the frame rate was set at 6 fps for imaging of all the groups.

### Electroretinogram analysis

Eyes of rd12 mice were mock injected or with AAV2 vectors via subretinal route with a 33 gauge blunt needle. Scotopic electroretinogram (ERG) was measured 10 weeks after gene transfer, and after a dark-adaptation overnight. ERG was recorded as per the manufacturer's instruction (Phoenix Research Lab). Briefly, mice were anesthetized with an intraperitoneal injection of ketamine (80 mg/kg) and xylazine (12 mg/kg), followed by pupil dilation by phenylephrine+tropicamide (Sunways India Pvt. Ltd.). Mice were placed on a heating pad and the reference electrode was subcutaneously placed under the forehead between the ears, whereas the ground electrode was placed under the tail subcutaneously. Corneal electrode was placed on the cornea after applying 2.5% Hypromellose (OCuSOFT, Rosenberg, NC). ERG was recorded with the intensity of light flash varying between −1.7 and 3.1 log cd s/m^2^.

### Statistical analysis

Statistical analysis was performed by either Student's *t*-test or ANOVA by GraphPad Prism 7 (GraphPad, La Jolla, CA) as applicable. Values obtained between the test and control groups were considered to be statistically significant if the *p*-value was <0.05. *p*-Values at various confidence intervals are denoted as *p < 0.05, **p < 0.01, and ***p < 0.001.

## Results

### Inhibition of host cellular Neddylation improves transduction of AAV2 vectors *in vitro*

To determine the role of cellular Neddylation during AAV2 infection, we used a small-molecule inhibitor of Neddylation, MLN4924. MLN4924 prevents assembly of NEDD8 and Neddylation activating enzyme (NAE1) protein complex.^37^ Since the role of SUMOylation by siRNA targeting is previously established,^29^ we focused on cellular Neddylation process. For our initial studies, we pretreated human cervical carcinoma cells (HeLa) with MLN4924 at 4.7 nM, 470 nM, and 1 μM of for 3 h, followed by infection with scAAV2-EGFP vectors. In this case, even at lower concentrations of MLN4924, transduction potential of AAV2 vectors were improved by ∼45% (47.76% [4.7 nM], 46.3% [470 nM], 47.41% [1 μM] vs. 32.41% [AAV2 alone]) (Supplementary Fig. S1). This suggests that cellular Neddylation could be a rate-limiting step during AAV2 transduction. Further studies such as specific targeting of Neddylation pathway genes by RNA interference are warranted to understand the molecular mediators of this process.

### Specific genes in the Neddylation and SUMOylation pathway are dysregulated upon AAV2 infection

To determine the changes to Neddylation and SUMOylation machinery during AAV2 infection, transcript levels of Neddylation and SUMOylation pathway-specific genes were measured,^21,38^ including the activating enzyme (E1), a conjugating enzyme (E2), and the NEDD8 and SUMO-1 gene that are known to be important in regulating this pathway. Since multiple ligating enzymes (E3) are known to be important for both Neddylation and SUMOylation in a substrate-specific manner,^21,38^ the mRNA level for E3 enzymes was not assessed. We performed a time-course analysis of the target genes in HeLa cells mirroring different stages of AAV2 infection (30 min, 2, 6, 12, and 24 h). Our data show that Neddylation genes such as *APPBP1* (2.78-fold), *UBA3* (5.49-fold), *UBC12* (2.31-fold) and SUMOylation genes such as *SAE1* (4.69-fold), *SAE2* (3.12-fold), *UBC9* (2.22-fold), *SUMO1* (6.36-fold) were significantly upregulated as early as the 2-h time point after infection (Supplementary Fig. S2a, b). Several studies^14,39^ have indicated that AAV2 undergoes cytoplasmic trafficking during this time point in HeLa cells, and thus, it is conceivable that Neddylation and SUMOylation signaling pathways are specifically activated upon AAV2 infection *in vitro*.

### AAV2 mutant vectors modified at Neddylation and SUMOylation sites demonstrate an increase in transduction efficiency *in vitro*

**Figure 1.**
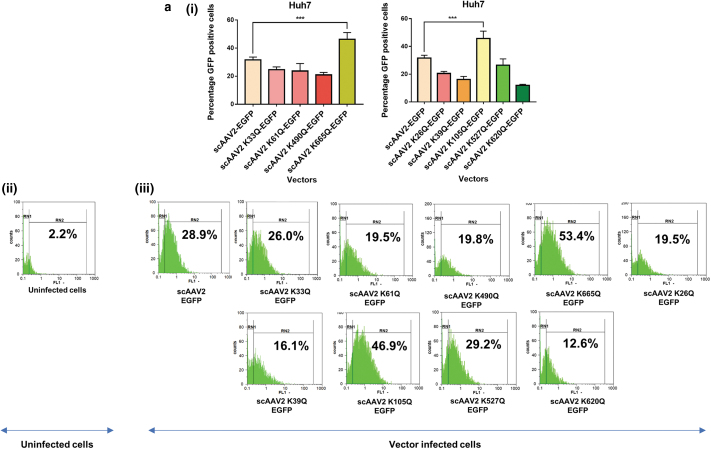

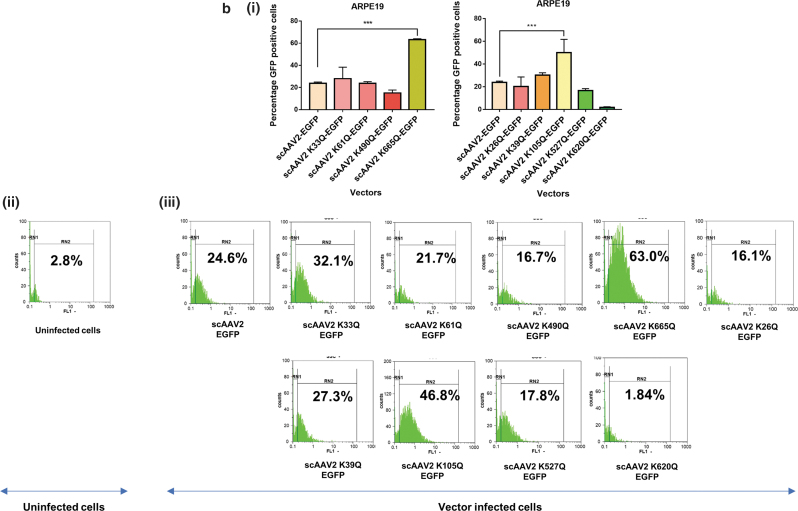

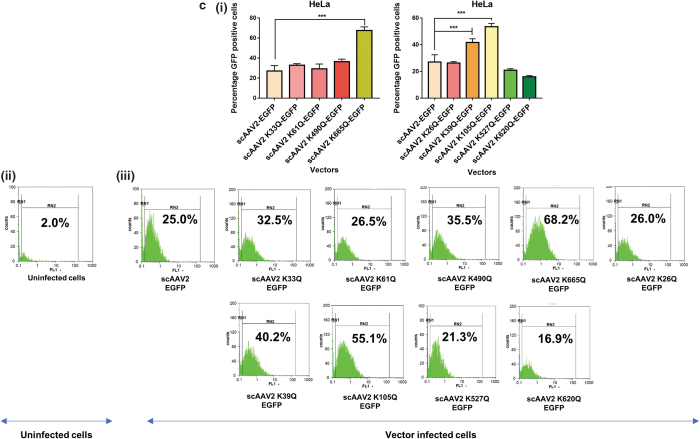
Transduction efficiency of AAV2 mutant vectors *in vitro*. About 3 × 10^4^ Huh7, ARPE19, or HeLa cells were mock-infected or infected with scAAV2-EGFP and scAAV2-EGFP mutant vectors for 3 h. Forty-eight hours later, the transgene (GFP) expression was measured by flow cytometry (CyFlow, Sysmex-Partec, Kobe, HP, Japan). Quantitative data for Neddylation or SUMOylation target-site mutants **(i)** and representative histograms **(ii, iii)** in Huh7 cells **(a)**, ARPE19 cells **(b)**, or HeLa cells **(c)** are shown. scAAV2-EGFP control was shared between Neddylation and SUMOylation mutants. Dunnett's multiple comparisons test was used to determine the statistical significance. Data are expressed as mean ± SD, *n* = 4, ***p ≤ 0.001. Data presented are a representative set from two independent biological replicate analysis. Values on histograms **(ii, iii)** have been manually shown for one replicate sample from entire analysis. The MFI (mean fluorescence intensity) for the same samples are shown in Supplementary Fig. S3. AAV, adeno-associated virus; ARPE, adult retinal pigmental epithelium; EGFP, enhanced green fluorescent protein; sc, self-complementary. Color images are available online.

We shortlisted the top five residues in AAV2 capsid predicted to be Neddylated by the software NeddyPreddy and the top five consensus SUMOylation targets identified by both GPS-SUMO and SUMOplot analysis (Supplementary Tables S6 and S7) for further site-directed mutagenesis. These included lysine residues at AAV2-K33, K61, K490, K640, K665 for Neddylation and AAV2-K26, K39, K105, K527, K620 residues for SUMOylation. These amino acids were mutated to corresponding glutamine (K>Q) residues. The average viral titers for these mutants were not significantly different from wild-type vectors (Supplementary Tables S8 and S9). We then assessed the transduction efficiency of these mutant vectors at an MOI of 5×10^3^ in multiple cell lines, including Huh7, ARPE19, and HeLa cells. Flow cytometry analysis showed a significantly higher increase in EGFP gene expression from Huh7 cells infected with scAAV2 K105Q mutant vector (45.7% ± 5.3% vs. 31.64% ± 2.0%) and scAAV2 K665Q mutant vector (46.28% ± 4.49% vs. 31.64% ± 2.0%) in comparison to WT-AAV2 vector–infected Huh7 cells (Fig. 1a). Similarly, in ARPE19 cells, we observed a significantly higher increase in EGFP expression from cells infected with scAAV2 K105Q mutant vector (50.11% ± 11.6% vs. 23.82% ± 1.14%) and scAAV2 K665Q mutant vector (63.16% ± 0.84% vs. 23.82% ± 1.14%) in comparison to WT-AAV2 vectors (Fig. 1b). As shown in Fig. 1c, the K105Q (53.48% ± 2.44% vs. 27.01% ± 5.5%) and K665Q (67.62% ± 3.57% vs. 27.01% ± 5.5%) mutants demonstrated a similar increase in transgene expression in HeLa cells, suggesting that these mutant vectors had higher infectivity consistently in multiple cell types. Also, the mean fluorescence intensities for scAAV2 K105Q and scAAV2 K665Q mutant vectors were also improved in comparison to cells infected with scAAV2-EGFP vector in all the cell lines tested (Supplementary Fig. S3). The infectivity of the vector K640Q, was modest and less than that of WT-AAV2 vectors (data not shown). Interestingly, in a previous study from our group, two sites utilized here (K490Q, Neddylation and K527Q, SUMOylation) were also predicted to affect ubiquitination by UbiPred tool and thus had been mutated to corresponding R residues (K490R, K527R).^40^ The transduction efficiency from both these forms of mutant vectors carrying K > R and K > Q substitutions is similar in the HeLa cells, thus pointing to the equivalence of these modifications. We further assessed the rate of viral entry and our results show that the entry profile of K105Q and K665Q mutants was similar to that of WT-AAV2 vectors (Supplementary Fig. S4). Therefore, the increased transduction seen with K105Q and K665Q mutants may be due to a decreased SUMOylation and Neddylation of AAV2 capsid, during its packaging or viral trafficking.

### AAV2 mutant capsid modified at a SUMOylation site demonstrates reduced levels of SUMO-1 protein

**Figure 2.**
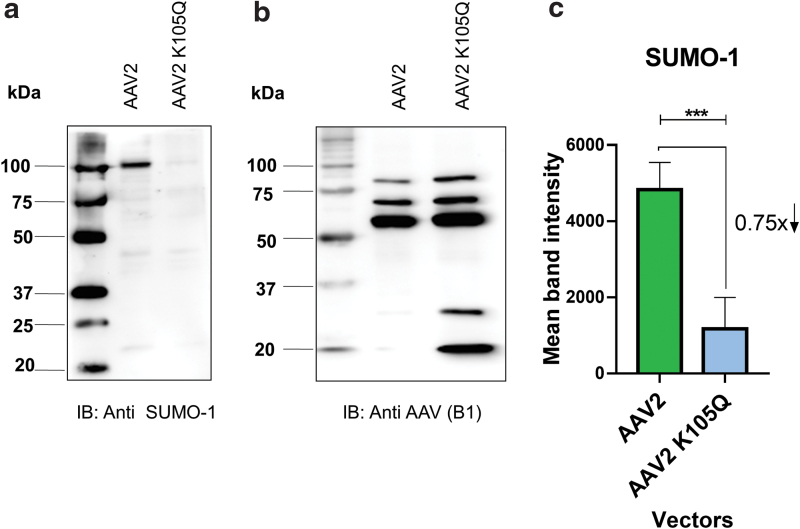
Western blot analysis of AAV2 vectors. About 1.42 × 10^10^ vgs of AAV2 and AAV2 K105Q vectors were resolved by denaturing SDS-PAGE. The level of SUMO-1 protein on vector capsids **(a)** were quantified **(c)** as described in the Materials and Methods section. Anti-AAV capsid B1 antibody was used as a loading control **(b)**. The field of view pertaining to loaded samples from the entire gel is shown in the image. Exposure time for SUMO-1 and B1 antibody immune-reactive blots was 2 min 49 s and 36 s, respectively. Data presented are representative set from three independent biological replicates. Paired *t*-test was performed to determine the statistical significance. Data are expressed as mean ± SD, *n* = 3, ***p ≤ 0.001. SDS-PAGE, sodium dodecyl sulfate–polyacrylamide gel electrophoresis; SUMO, small ubiquitin-like modifier; vgs, vector genomes. Color images are available online.

Since the SUMOylation site–modified (K105Q) AAV2 vector had a consistent increase in transduction in multiple cell lines, we further wished to determine the mechanistic basis of this phenotype. We thus assessed the levels of SUMO-1 protein in the freshly packaged AAV2-K105Q and AAV2 wild-type vectors by immunoblotting (Fig. 2). Our data shows that the AAV2 K105Q vector had a significantly lower amount (∼75%) of SUMOylation in comparison to AAV2 wild-type vectors. This observation also confirms that K105 site is a major target of SUMOylation in AAV2 capsid.

### Neddylation site–modified AAV2 vector has minimal levels of NEDD8 marker protein on viral capsid

In our next set of experiments, we wished to understand the Neddylation profile of freshly packaged AAV2-K665Q vector in comparison to wild-type AAV2 vectors. Since the level and abundance of Neddylation was very low on AAV2 wild-type vectors, which was not detected by standard Western blot analysis (data not shown), we performed a dot blot assay to analyze the Neddylation levels on total viral capsid proteins. As shown in Supplementary Fig. S5, the AAV2 mutant (K665Q) had modestly reduced levels (−17.77% ± 9.51%) of NEDD8 protein when compared with AAV2 WT vector (Supplementary Fig. S5). These data suggest that K665Q site in AAV2 capsid is a target for cellular Neddylation during vector packaging.

### Neddylation and SUMOylation site–modified AAV2 vectors improve circulating levels of coagulation factor IX in hemophilia B mice

**Figure 3.**
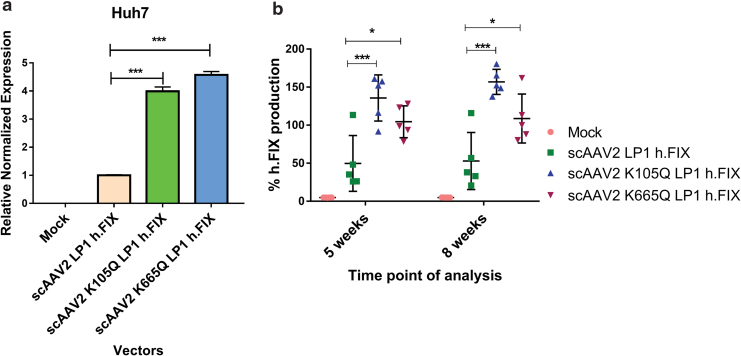
Efficiency of Neddylation or SUMOylation site–modified AAV2 vectors for gene transfer into hepatic cells *in vitro* or in a murine model of hemophilia B. **(a)** Human factor IX (h.FIX) transcript levels assessed by quantitative PCR from Huh7 cells infected with scAAV2-h.FIX wild type or mutant vectors are shown. **(b)** Levels of h.FIX in plasma were determined 5 and 8 weeks after injection of 5 × 10^10^ vgs of AAV2 vectors per animal. Dunnett's multiple comparisons test was used to determine the statistical significance. Data are expressed as mean ± SD (*n* = 5 animals per experimental group). ***p ≤ 0.001, *p ≤ 0.05. PCR, polymerase chain reaction. Color images are available online.

**Figure 4.**
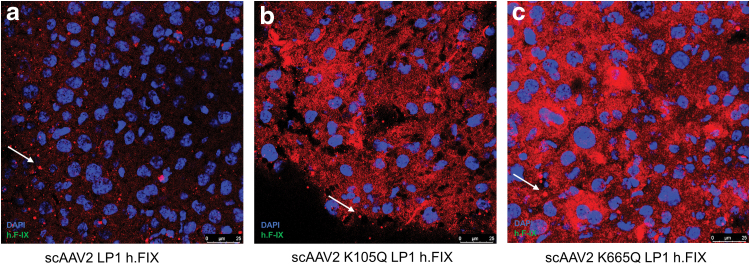
Immunohistochemistry for human factor IX after hepatic gene transfer *in vivo*. Human factor IX (h.FIX) expression was detected by fluorescence microscopy 9 weeks postinjection of 5 × 10^10^ vgs/animal of scAAV2 LP1 h.FIX **(a)**, scAAV2 K105Q LP1 h.FIX **(b)**, or scAAV2 K665Q LP1 h.FIX **(c)**. Representative images are shown. Multiple bright nonspecific fluorescent spots were detected (marked by *arrow*) surrounding specific signals during imaging. Original magnification 400 × . Color images are available online.

In anticipation of testing the *in vivo* efficacy of AAV mutant vectors developed here, we first prescreened the scAAV2 wild type and the K105Q and K665Q vectors expressing h.FIX in Huh7 cells. As shown in Fig. 3a, the K105Q and K665Q vectors demonstrated a 3.9- to 4.5-fold increase in h.FIX transcript levels, respectively, which further confirmed our findings with the reporter GFP transgene (Fig. 1). We then investigated the therapeutic potential of K105Q and K665Q vectors in a preclinical model of hemophilia B. Groups of hemophilia B mice (*n* = 5 mice per group), were mock (PBS)-injected or injected AAV2 wild type, K105Q and K665Q mutant vectors expressing h.FIX at a dose of 5 × 10^10^ vgs/animal. The h.FIX antigen levels were assessed 5 and 8 weeks, postvector administration by h.FIX-specific ELISA. At the 5 weeks' time point, the mean h.FIX levels in animals that received the mutant K105Q vector were 135.8% ± 30.37% and for K665Q mutant it was 104.5% ± 20.9%, whereas in animals that received the wild-type vectors, the h.FIX levels were 49.83% ± 36.66%. After 8 weeks, h.FIX levels were near normal levels in animals that received mutant AAV2 (156.9% ± 16.46% [K105Q] and 108.8% ± 32.18% [K665Q]) when compared with wild-type AAV2 vector–injected animals (52.92% ± 37.54%, Fig. 3b). These data were further corroborated by immunostaining, which showed a higher h.FIX expression in the liver sections of K105Q and K665Q mutant–administered mice when compared with AAV2-administered mice (Fig. 4). These findings highlight that a single injection of Neddylation and SUMOylation mutant AAV2-h.FIX vectors can generate physiological levels of h.FIX and further confirm their therapeutic potential for hepatic gene therapy of hemophilia B.

### Neddylation and SUMOylation mutant vectors are not immunogenic in comparison to wild-type AAV2 vectors

In our next set of studies, we characterized the immune profile of the mutant AAV2-h.FIX vectors 9 weeks after hepatic gene transfer in hemophilia B mice. As shown in Supplementary Fig. S6, we did not observe a significant increase in the subpopulation of T cells including *T helper cells* (14.67% ± 2.75% [AAV2] vs. 15.32% ± 3.08% [K105Q], 14.05% ± 2.91% [K665Q]), *cytotoxic T cells* (14.59% ± 2.07% [AAV2] vs. 18.3% ± 7.22% [K105Q], 14.68% ± 3.62% [K665Q]), or *regulatory T cells* (1.70% ± 0.38% [AAV2] vs. 1.82% ± 0.76% [K105Q], 1.35% ± 0.34% [K665Q]) between the mutant- and wild-type AAV2 vector–administered hemophilia B animals. A similar data were obtained when the B cells were enumerated (Supplementary Fig. S6d).

Furthermore, we harvested splenocytes from the mock-treated and AAV2-treated mice and the capsid-specific CD8^+^ T cell-based response was evaluated by the IFN-γ ELISPOT assay. Our data shown in Supplementary Fig. S7 demonstrate that concanavalin A (positive control) generated 1026 ± 303 number of spots per million stimulated splenocytes. Among the test groups, the IFN-γ response from splenocytes in animals that had gene transfer was at basal levels and was not significantly different between splenocytes of mice that received wild-type AAV2 or mutant AAV2 vectors. These data suggest that in murine models of hemophilia B, the host T cell response against AAV2 vectors is negligible after a single low dose of AAV2 vectors as demonstrated earlier.^5^

### SUMOylation site–modified (K105Q) AAV2 vector improves transgene expression after ocular gene transfer in mouse retina

**Figure 5.**
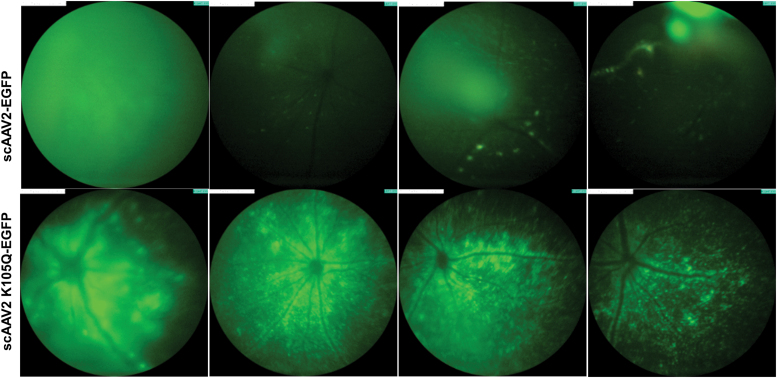
Ocular gene transfer in C57BL6/J mice with SUMOylation site–modified K105Q vector. A fundus imaging of murine eyes was performed 4 weeks after administration of AAV2 wild-type and AAV2 K105Q vectors. For these experiments, we used a Micron IV imaging system (Phoenix Research Laboratories, Pleasanton, CA) that employs a standard mouse objective and a field of view at 50° (1.8 mm diameter). Intensity was set at maximum and gain was set at 15 db; the frame rate was set at 6 fps for imaging of all the groups. Image analysis was performed by using Concentric Circle Plugin in the ImageJ software, and AAV2 K105Q group had a 1.57-fold higher EGFP expression in comparison to AAV2 wild-type eyes (*n* = 4 to 7 eyes). Representative images are shown. Color images are available online.

To further assess the gene transfer potential of mutant vectors in another tissue type, we chose the AAV2 SUMOylation site–modified vector, K105Q, that had significantly better hepatic transduction *in vivo* (Fig. 3b). Both the AAV2 wild type and K105Q mutant vector expressing a reporter gene, EGFP, were injected intravitreally in 1 μL volume at a dose of 3 × 10^8^ vgs/eye (*n* = 4 to 7 eyes per group) in normal C57BL6/J mice. Four weeks after intraocular gene transfer, we observed a 1.57-fold increase in EGFP expression in AAV2-K105Q mutant–administered mice in comparison to AAV2 wild-type vector–administered mice (Fig. 5). It should be noted that some of the AAV2 and AAV2 K105Q vector–injected eyes had variable EGFP expression, which is possibly due to technical limitations in the manual injection procedure. Nonetheless, our data underscore the potential of AAV2 K105Q vector for therapeutic ocular gene transfer.

### AAV2-K105Q vectors encoding RPE65 demonstrate phenotypic correction after subretinal gene transfer in rd12 mice

**Figure 6.**
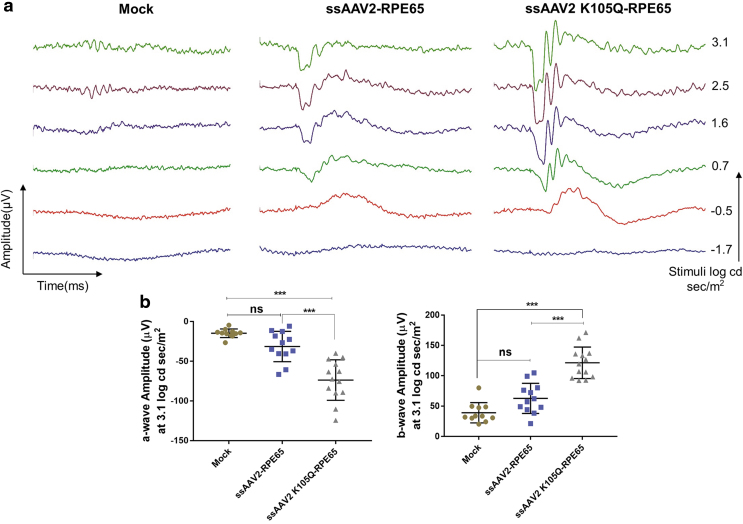
SUMOylation site–modified vectors expressing RPE65 demonstrate phenotypic rescue in rd12 mice model. Eyes of rd12 mice were mock-injected or injected with ssAAV2-RPE65 and ssAAV2K105Q-RPE65 vectors at a dose of 7 × 10^8^ vectors via subretinal route. Scotopic ERG recordings were performed 10 weeks postvector administration and their representative image **(a)** and quantification data **(b)** are shown. Completely opaque eyes caused by injury were eliminated from the recording data set. Dunnett's multiple comparisons test was used to determine the statistical significance. Data are expressed as mean ± SD (*n* = 11–13 eyes per experimental group). ***p ≤ 0.001. ERG, electroretinogram. Color images are available online.

To further evaluate the therapeutic efficiency of AAV2-K105Q vectors for ocular gene therapy, we administered AAV2 wild type and K105Q expressing human retinal pigmental epithelium gene encoding 65 kDa protein (RPE65) in groups of rd12 mice. Approximately 1–2 μL of vectors containing 7 × 10^8^ vgs was administered by subretinal route into the eyes of rd12 mice. The phenotypic rescue was measured by ERG analysis 10 weeks after vector administration. Representative ERG waveforms are shown in Fig. 6a. The A-wave amplitude for K105Q vector–administered mice was significantly elevated to −73.68 ± 25.49 μV in comparison to eyes that received AAV2 wild-type vectors (−31.43 ± 19.09 μV) and mock-injected animals (−14.72 ± 5.43 μV) (Fig. 6b). The B-wave amplitude for AAV2 K105Q–administered and AAV2 wild type–administered eyes was 121.4 ± 25.92 and 62.75 ± 24.82 μV, respectively (Fig. 6b). This highlights that ocular gene therapy with AAV2 K105Q-RPE65 vectors has a therapeutic A-wave amplitude response (*p* < 0.001) and B-wave response (*p* < 0.001) when compared with rd12 mice that received wild-type AAV2 vectors.

## Discussion

AAV-based vectors are a valuable tool in the field of gene therapy, particularly for gene transfer into postmitotic tissues such as the liver or eye. Nonetheless, since host immune and transduction barriers to AAV vectors remain,^5,16^ we^40^ and several others^16^ have employed multiple strategies to overcome them. In addition, technologies to improve the vector efficiency such as pseudopackaging of AAV genomes,^41,42^ insertional mutation strategies to enhance efficiency,^16,43^ and use of protease-activatable AAV-based vector systems^44^ are available. The goal of each of these strategies is to target the underlying molecular mechanisms responsible for the inhibition of AAV transduction or intracellular events that lead to degradation of capsid proteins and its interplay with immune effectors. PTMs involving ubiquitination and phosphorylation on AAV capsid have already been investigated,^15^ and more recently, acetylation of several AAV serotypes during the packaging process has been reported.^45^ Among the plethora of PTMs not studied, we have analyzed the impact of UBLs such as Neddylation and SUMOylation on AAV2, due to its distinct yet functional overlap with the ubiquitination pathway.^46,47^

Neddylation has been demonstrated to have a regulatory role in host–viral interactions in a context-dependent manner.^48,49^ In the case of influenza A virus 9, Neddylation of polymerase basic protein 2 (PB2) inhibits its replication through E3 ligase HDM2. Further *in vitro* studies by mutation of a residue responsible for Neddylation in PB2 increased the stability of PB2 and enhanced the viral replication.^48^ Hughes *et al.* demonstrated that Neddylation is an essential process required for maintaining Kaposi's sarcoma-associated herpes virus latency.^49^ Similarly, SUMOylation is known to have multiple roles during viral infection of a host cell. A recent study used siRNA-mediated knockdown of SAE1, SAE2, and UBC9 enzymes of SUMOylation pathway in HeLa cells and concluded that SUMOylation negatively regulates AAV transduction process irrespective of single-stranded or self-complimentary genomes and the dosage of AAV used.^29^ The role of SUMOylation in the context of life cycle of other viruses such as adenovirus, HSV-1, and Epstein-Barr virus (EBV) has also been established.^27^ Viruses such as HSV-1 and EBV utilize the SUMOylation process to maintain their persistence with host cells,^27^ whereas host cellular protein SUMOylation events confers specific immune targeting of viruses such as human cytomegalovirus and lentivirus.^50^

Considering the wide-ranging implications of both Neddylation and SUMOylation modifications in case of several viruses and their host, we further set out to determine its impact on AAV-mediated gene delivery. Since the role of SUMOylation pathway in AAV2 transduction is known,^29^ we first focused our efforts on deducing the role of Neddylation pathway. In our initial studies, we identified that AAV2 infection of HeLa cells upregulates crucial mediators of (*APPBP1*, *UBA3*, *UBC12*) of Neddylation signaling pathway. Conversely, the inhibition of Neddylation by a small-molecule inhibitor, MLN4924, at various concentrations (4.7 nM, 470 nM, and 1 μM; Supplementary Fig. S1) improved AAV2 transduction by ∼45%. These data suggest that cellular Neddylation status could regulate AAV2 vector transduction. These data mirror previous studies^15^ that established the role of ubiquitination in AAV2 life cycle.^15^ We speculate that infection of AAV2 triggers intracellular Neddylation machinery and may further lead to the ubiquitination of the viral capsid. However, further studies are needed to understand this phenomenon. Interestingly, interdependency of ubiquitination and Neddylation has been shown in the case of p53, which was either ubiquitinated or Neddylated by the same E3 ligase (MDM2) under different cellular conditions.^51^

Our observation here that the NEDD8 inhibitor improves AAV2 transduction and previous observations that siRNA against SUMOylation pathway have a similar effect *in vitro*^29^ presents a possibility of coadministration of small-molecule inhibitors to Neddylation or SUMOylation during gene transfer with AAV2 vectors to improve its efficiency. However, both Neddylation and SUMOylation are crucial factors for the maintenance of normal cellular physiology, and thus, this approach is likely to lead to severe side effects.^52,53^ Thus, we preferred to modify the AAV2 capsid residues that are potential substrates for Neddylation or SUMOylation and as determined by prediction algorithms.^30,31^

Among the 10 different Neddylation and SUMOylation site mutant vectors developed, K105Q and K665Q showed a significant higher transgene expression in Huh7 cells (Fig. 1). To understand if this increase is due to an improved cellular uptake of the mutant vectors, we performed a viral entry assay. The entry profile of K105Q and K665Q mutants was similar to WT-AAV2 vectors (Supplementary Fig. S5). This suggests that modulation of intracellular mechanisms such as Neddylation or SUMOylation is more likely to lead to enhanced transduction as seen with our mutant vectors. This is further supported by our immunoblotting studies with AAV2 K105Q or K665Q mutant (Fig. 2 and Supplementary Fig. S4), which showed significantly lower level of SUMO-1 or NEDD8 protein on its capsid. However, it is plausible that only a fraction of packaged capsids is SUMOylated or Neddylated at the VP monomer level.

To further understand if these modified vectors have therapeutic potential, we performed hepatic gene transfer of AAV2-K105Q and AAV2-K665Q vectors packaged with human coagulation h.FIX in a murine model of hemophilia B. Both the mutant vectors demonstrated a significant increase in h.FIX levels in hemophilia B mice. Two months after gene transfer, the h.FIX levels in AAV2-K105Q mutant and the AAV2-K665Q remained significantly higher when compared with animals that were administered with wild-type AAV2 vector. Additionally, these mutant AAV2 vectors had an immune profile similar to wild-type AAV2 vectors (Supplementary Figs. S6 and S7). Taken together, these findings suggest that it is possible to achieve physiological levels of h.FIX expression at very low doses of AAV2-K105Q and AAV2-K665Q vectors, without activating the cellular immune response *in vivo*. Subsequently, we expanded the scope of our preclinical evaluation of the novel UBL site–modified AAV vectors by ocular gene therapy in a murine model of a retinal degenerative disease. A comprehensive analysis of the K105Q mutant AAV2 vectors was performed *in vivo*, with two transgenes (*GFP and RPE65*) and two routes of vector administration (*intravitreal* and *subretinal*). Our results showed that the intravitreal administration of an AAV2 (K105Q) vector containing a reporter gene had a significant increase in GFP expression in the retina of treated mice, 4 weeks after gene transfer. We then evaluated the potential of the most consistent mutant, AAV2-K105Q vector, with a therapeutic transgene (RPE65) in LCA2 mice. Ten weeks after subretinal gene transfer, a partial visual correction was observed in mice that received gene therapy, by ERG analysis.

This study also has some limitations. The mechanistic basis of the improved transduction of SUMOylation and Neddylation site–modified vectors needs to be ascertained in detail, including the abundance of these modifications at the VP capsid monomer level. In addition, a majority of alternate AAV1–10 serotypes have conserved Neddylation and SUMOylation sites, and thus, it remains to be seen if such modifications in other serotypes are beneficial. Furthermore, a long-term follow-up of hemophilia B or rd12 mice treated with the mutant AAV2 vectors will be required to assess their safety profile, comprehensively.

In conclusion, our study has unraveled the hitherto unknown roles of Neddylation and SUMOylation during AAV2 infection. Furthermore, the development of Neddylation and SUMOylation target-site–engineered AAV2 vectors may be beneficial for therapeutic hepatic and ocular gene transfer.

## Supplementary Material

Supplemental data

Supplemental data

Supplemental data

Supplemental data

Supplemental data

Supplemental data

Supplemental data

Supplemental data

Supplemental data

Supplemental data
