# Supplementary material for: Rational Engineering and Preclinical Evaluation of Neddylation and SUMOylation Site Modified Adeno-Associated Virus Vectors in Murine Models of Hemophilia B and Leber Congenital Amaurosis
Source: Hum Gene Ther. 2019 Nov 26;30(12):1461–76. doi: 10.1089/hum.2019.164 (PMC6919284; doi:10.1089/hum.2019.164)
Supplement: Supplemental data [file Supp_Tables1-2.pdf]

Supplementary Table S1. *Primers designed for analyzing gene expression of Neddylation pathway genes*

Pathway Enzyme	*Name*	*Primer*	*Length (bp)*	*Amplicon Length (bp)*
Neddylation				
E1	APPBP1	5′-CTTCCTTCAAAGAAGCAGTATCGG-3′	24	139
		5′-CTGGACTCTCTTCCACAAAACTTC-3′	24	
E1	UBA3	5′-GGTCGCTGGAACCATGTAAAGA-3′	22	129
		5′-GCCAGCTCCAATGACTAGAACTTT-3′	24	
E2	UBC12	5′-TCCAGAAGGACATAAACGAGCTG-3′	23	97
	(UBE2M)	5′-GGACAGATGACCAGCTTGAAGT-3′	22	
	NEDD8	5′-GAACCTACAGACAAGGTGGAGC-3′	22	119
		5′-GCTGCTGTCTTCTCATCATTCATC-3′	24	

Supplementary Table S2. *Primers designed for analyzing gene expression of SUMOylation pathway genes*

Enzyme Targets in	*Name*	*Primer*	*Length (bp)*	*Amplicon Length (bp)*
SUMOylation pathway				
E1	SAE1	5′-AGTCATTTTTCACTCAATTCGATGCT-3′	26	122
		5′-GCCAAAAACATCTCCTGTAAAGAAC-3′	25	
E1	SAE2	5′-GCTGGGTATCTTGGACAAGTAACT-3′	24	92
		5′-CCAGGAAAGGTTCTCTGGGTC-3′	21	
E2	UBC9	5′-GCCATTCCAGGAAAGAAAGGGA-3′	22	90
	(UBE2I)	5′-TGGTGGCGAAGATGGATAATCATC-3′	24	
	SUMO1	5′-GGCAAAAACCTTCAACTGAGGAC-3′	22	91
		5′-GTGAATCTCACTGCTATCCTGTCC-3′	24	
