# Supplementary material for: Rational Engineering and Preclinical Evaluation of Neddylation and SUMOylation Site Modified Adeno-Associated Virus Vectors in Murine Models of Hemophilia B and Leber Congenital Amaurosis
Source: Hum Gene Ther. 2019 Nov 26;30(12):1461–76. doi: 10.1089/hum.2019.164 (PMC6919284; doi:10.1089/hum.2019.164)
Supplement: Supplemental data [file Supp_Tables3-6.pdf]

Supplementary Table S3. *Neddylation sites predicted by NeddyPreddy on AAV2 VP1 capsid protein*

S. No.	*Position*[a]	*Peptide*	*Score*	*Probability*
1	33	23-WKLKPGPPPP**K**PAERHKDDSR-43	1.09	0.53
2	640	630-PSPLMGGFGL**K**HPPPQILIKN-650	0.85	0.40
3	61	50-KYLGPFNGLD**K**GEPVNEADAA-70	0.66	0.30
4	490	480-GPCYRQQRVS**K**TSADNNNSEY-500	0.43	0.21
5	665	655-ANPSTTFSAA**K**FASFITQYST-675	0.36	0.18
6	39	29-PPPPKPAERH**K**DDSRGLVLPG-49	0.18	0.13
7	24	14-TLSEGIRQWW**K**LKPGPPPPKP-34	0.16	0.13
8	161	151-VEPDSSSGTG**K**AGQQPARKRL-171	0.03	0.10
9	620	610-DVYLQGPIWA**K**IPHTDGHFHP-630	0.03	0.10

The predicted site is presented in bold font.
[a]The numbering of amino acids in VP1 capsid protein is according to the NCBI Protein ID: YP_680426.1.

Supplementary Table S4. *SUMOylation sites predicted by GPS-SUMO on AAV2 VP1 capsid protein*

S. No.	*Position*[a]	*Peptide*	*Score*
1	105	98-AEFQER**LKED**TSFGG-112	37.13
2	137	130-GLVEEP**VKTA**PGKKR-144	38.488
3	527	520-GPAMAS**HKDD**EEKFF-534	42.941

Indicated region denotes consensus motif for SUMOylation.
[a]The numbering of amino acids in VP1 capsid protein is according to the NCBI Protein ID: YP_680426.1.

Supplementary Table S5. *SUMOylation sites predicted by SUMOplot on AAV2 VP1 capsid protein*

S. No.	*Position*[a]	*Peptide*	*Score*
1	105	99-EFQER **LKED** TSFGG-112	0.91
2	640	634-MGGFG **LKHP** PPQIL-647	0.8
3	26	20-RQWWK **LKPG** PPPPK-33	0.73
4	620	614-QGPIW **AKIP** HTDGH-627	0.69
5	527	521-PAMAS **HKDD** EEKFF-534	0.52
6	39	33-KPAER **HKDD** SRGLV-46	0.52
7	544	538-GVLIF **GKQG** SEKTN-551	0.5
8	314	308-PKRLN **FKLF** NIQVK-321	0.5
9	161	155-SSSGT **GKAG** QQPAR-168	0.5
10	61	55-PFNGL **DKGE** PVNEA-68	0.5
11	143	137-KTAPG **KKRP** VEHSP-150	0.37
12	532	526-HKDDE **EKFF** PQSGV-539	0.15

Indicated region denotes consensus motif for SUMOylation.
[a]The numbering of amino acids in VP1 capsid protein is according to the NCBI Protein ID: YP_680426.1.

Supplementary Table S6. *Primers designed to abolish predicted Neddylation sites from lysine to glutamine*

Mutation	*Primers*	*Size (bp)*
**K33Q**	5′-AAACCTGGCCCACCACCACCA**C**AGCCCGCAGAGCGGC ATAAGGA-3′<br>5′-TCCTTATGCCGCTCTGCGGGCT**G**TGGTGGTGGTGGGCC AGGTTT-3′	44
**K61Q**	5′-CTTCAACGGACTCGAC**C**AGGGAGAGCCGGTCAA-3′<br>5′-TTGACCGGCTCTCCCT**G**GTCGAGTCCGTTGAAG-3′	33
**K640Q**	5′-CATGGGTGGATTCGGACTT**C**AACACCCTCCTCCACAGAT-3′<br>5′-ATCTGTGGAGGAGGGTGTT**G**AAGTCCGAATCCACCCATG-3′	39
**K490Q**	5′-CCAGCAGCGAGTATCA**C**AGACATCTGCGGATAACAACAAC-3′<br>GTTGTTGTTATCCGCAGATGTCT**G**TGATACTCGCTGCTGG-3′	40
**K665Q**	5′-ACCACCTTCAGTGCGGCA**C**AGTTTGCTTCCTTC-3′<br>5′-GAAGGAAGCAAACT**G**TGCCGCACTGAAGGTGGT-3′	33

The boldface font indicates the site of mutation.
