# Supplementary material for: Rational Engineering and Preclinical Evaluation of Neddylation and SUMOylation Site Modified Adeno-Associated Virus Vectors in Murine Models of Hemophilia B and Leber Congenital Amaurosis
Source: Hum Gene Ther. 2019 Nov 26;30(12):1461–76. doi: 10.1089/hum.2019.164 (PMC6919284; doi:10.1089/hum.2019.164)
Supplement: Supplemental data [file Supp_Tables7-9.pdf]

Supplementary Table S7. *Primers designed for mutagenesis of predicted SUMOylation sites from lysine to glutamine residue*

Mutation	*Primer*	*Size (bp)*
**K26Q**	5′-TTTGGTGGTGGTGGGCCAGGTT**G**GAGCTTCCACCACTGT-3′<br>5′-ACAGTGGTGGAAGCTC**C**AACCTGGCCCACCACCACCAAA-3′	39
**K39Q**	5′-CCTGCTGTCGTCCT**G**ATGCCGCTCTGCGGGCTTT-3′<br>5′-AAAGCCCGCAGAGCGGCAT**C**AGGACGACAGCAGG-3′	34
**K105Q**	5′-CCCCAAAAGACGTATCTTCTT**G**AAGGCGCTCCTGAAACTCC-3′<br>5′-GGAGTTTCAGGAGCGCCTT**C**AAGAAGATACGTCTTTTGGGG-3′	41
**K527Q**	5′-AACTTTTCTTCATCGTCCT**G**GTGGCTTGCCATGGCCGGG-3′<br>5′-CCCGGCCATGGCAAGCCAC**C**AGGACGATGAAGAAAAGTT-3′	39
**K620Q**	5′-GTCCGTGTGTGGAATCT**G**TGCCCAGATGGGCCCCTGAAG-3′<br>5′-CTTCAGGGGCCCATCTGGGCA**C**AGATTCCACACACGGAC-3′	39

The boldface font indicates the site of mutation.

Supplementary Table S8. *Physical particle titers for the vectors (AAV2-EGFP and AAV2 Neddylation site mutants) generated in this study*

Vector	*Titer (vgs/mL)*
scAAV2-EGFP	2.00E+11
scAAV2 K33Q-EGFP	8.52E+11
scAAV2 K61Q-EGFP	4.31E+11
scAAV2 K490Q-EGFP	1.64E+12
scAAV2 K640Q-EGFP	1.56E+12
scAAV2 K665Q-EGFP	6.25E+11

The data are presented as vector genomes per milliliter (vgs/mL).

Supplementary Table S9. *Physical particle titers for the vectors (AAV2-EGFP and AAV2 SUMOylation site mutants) generated in this study*

Vector	*Titer (vgs/mL)*
scAAV2-EGFP	2.00E+11
scAAV2 K26Q-EGFP	8.71E+11
scAAV2 K39Q-EGFP	5.45E+11
scAAV2 K105Q-EGFP	4.69E+11
scAAV2 K527Q-EGFP	1.38E+12
scAAV2 K620Q-EGFP	4.14E+11

The data are presented as vector genomes per milliliter (vgs/mL).
