# Supplementary material for: Rational Engineering and Preclinical Evaluation of Neddylation and SUMOylation Site Modified Adeno-Associated Virus Vectors in Murine Models of Hemophilia B and Leber Congenital Amaurosis
Source: Hum Gene Ther. 2019 Nov 26;30(12):1461–76. doi: 10.1089/hum.2019.164 (PMC6919284; doi:10.1089/hum.2019.164)
Supplement: Supplemental data [file Supp_Fig1.pdf]

Supplementary Data

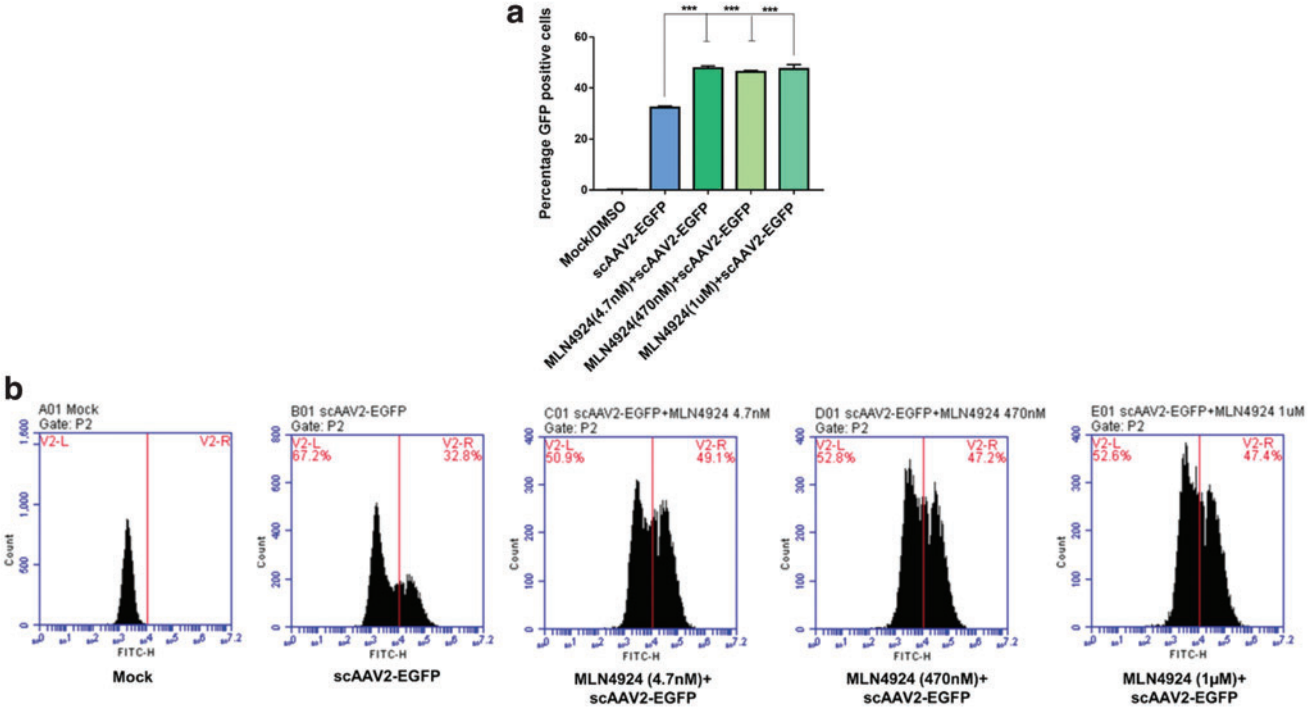

Supplementary Figure S1. Transduction efficiency of AAV2 vectors in cells pretreated with a small-molecule inhibitor of Neddylation. Approximately 3×10^4 HeLa cells were either mock-treated (DMSO) or treated with a Neddylation inhibitor, MLN4924, at a concentration of 4.7 nM, 470 nM, and 1 μM and incubated at 37°C for 3 h. After incubation, cells were infected with scAAV2-EGFP vectors at an MOI of 5×10^3 vgs/cell for 3 h. Forty-eight hours later, the transgene (GFP) expression **(a)** was quantified by flow cytometry (BD Accuri C6 Plus; BD Biosciences, Franklin Lakes, NJ). Data presented are a representative histogram **(b)** from a panel of five replicates for analysis carried out in **(a)**. Dunnett's multiple comparisons test was performed to determine the statistical significance. Data are expressed as mean ± SD, $n = 5$, $^{***}p \leq 0.001$. The experiments were repeated twice (biological replicate = 2) to confirm the findings. DMSO, dimethyl sulfoxide; GFP, green fluorescent protein; MOI, multiplicity of infection; SD, standard deviation.
