# Supplementary material for: Rational Engineering and Preclinical Evaluation of Neddylation and SUMOylation Site Modified Adeno-Associated Virus Vectors in Murine Models of Hemophilia B and Leber Congenital Amaurosis
Source: Hum Gene Ther. 2019 Nov 26;30(12):1461–76. doi: 10.1089/hum.2019.164 (PMC6919284; doi:10.1089/hum.2019.164)
Supplement: Supplemental data [file Supp_Fig2.pdf]

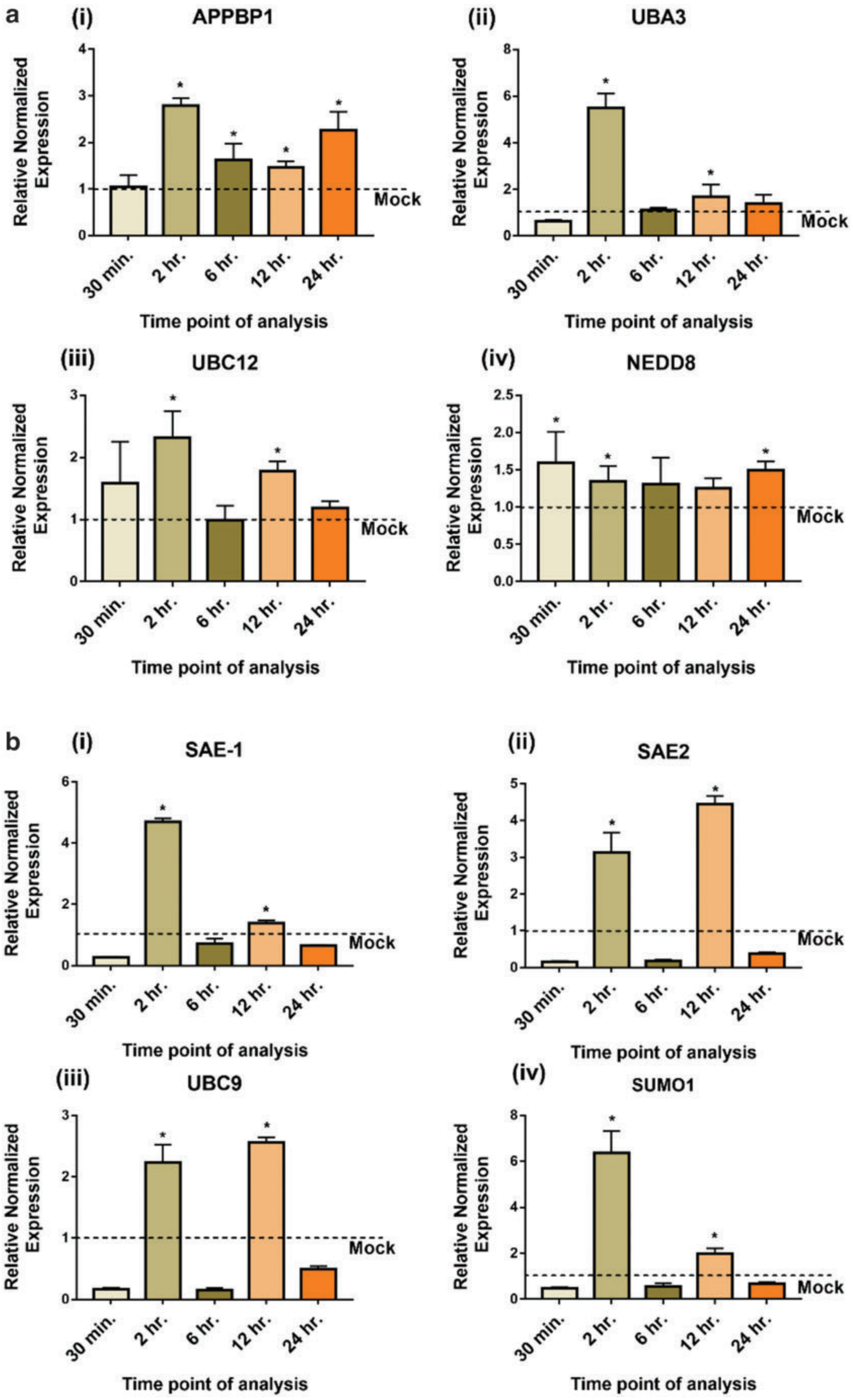


**Supplementary Figure S2.** Targeted transcript analysis of Neddylation and SUMOylation pathway genes in response to AAV2 infection. Approximately $8\times10^4$ HeLa cells were infected with scAAV2-EGFP vectors at an MOI of $5\times10^3$ vgs/cell. At various time points, total RNA from each of the treated condition in HeLa cells was isolated and transcript levels of Neddylation target genes **(a, i–iv)** and SUMOylation target genes **(b, i–iv)** were measured by qPCR. Beta-actin was used as an endogenous control for the normalization of data. Each time point had its own mock-treated control cells, and their transcript level is denoted by *dotted line*. Data presented are representative set from two independent biological replicates. Paired *t*-test was performed to determine the statistical significance. Data are expressed as mean ± SD, $n=3$, $*p\leq0.05$. qPCR, quantitative polymerase chain reaction.
