# Supplementary material for: Rational Engineering and Preclinical Evaluation of Neddylation and SUMOylation Site Modified Adeno-Associated Virus Vectors in Murine Models of Hemophilia B and Leber Congenital Amaurosis
Source: Hum Gene Ther. 2019 Nov 26;30(12):1461–76. doi: 10.1089/hum.2019.164 (PMC6919284; doi:10.1089/hum.2019.164)
Supplement: Supplemental data [file Supp_Fig3.pdf]

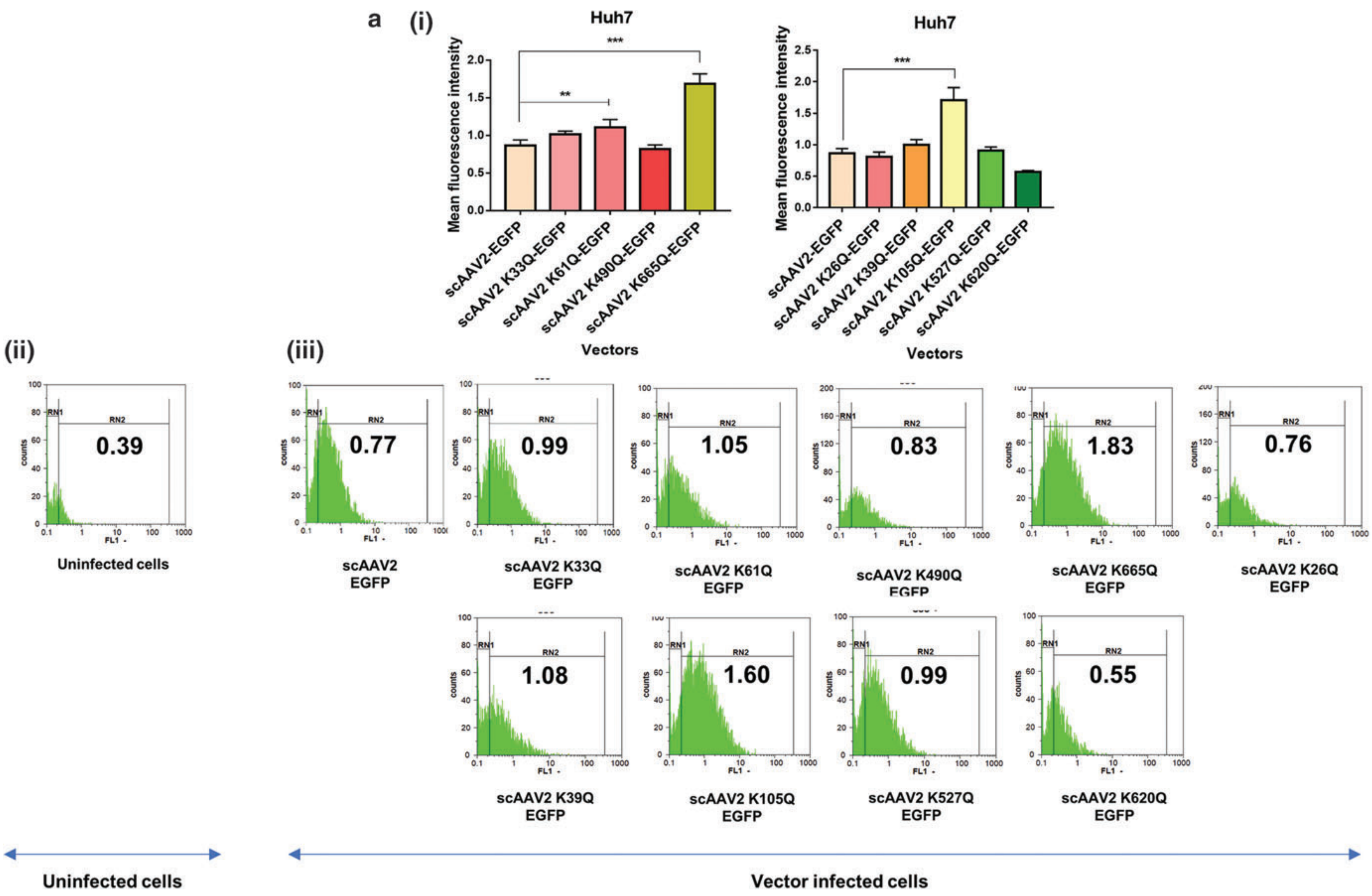

Supplementary Figure S3. MFI of AAV2 mutant vectors *in vitro*. The MFI for Neddylation or SUMOylation target-site mutants was quantified by flow cytometry (CyFlow, Sysmex-Partec, Kobe, HP, Japan) **(i)** and representative histograms **(ii, iii)** in Huh7 cells **(a)**, ARPE-19 **(b)**, or HeLa cells **(c)** are shown. scAAV2-EGFP control was shared between Neddylation and SUMOylation mutants. Dunnett's multiple comparisons test was used to determine the statistical significance. Data are expressed as mean ± SD, $n=4$, *$p \leq 0.05$, **$p \leq 0.01$, ***$p \leq 0.001$. Data presented are a representative set from two independent biological replicate analysis. MFI values on the histogram **(ii, iii)** have been manually shown for one replicate sample from entire analysis. The histogram plot shown here is similar to Fig. 1 that demonstrated the % GFP positivity for the same sample. MFI, mean fluorescence intensity.

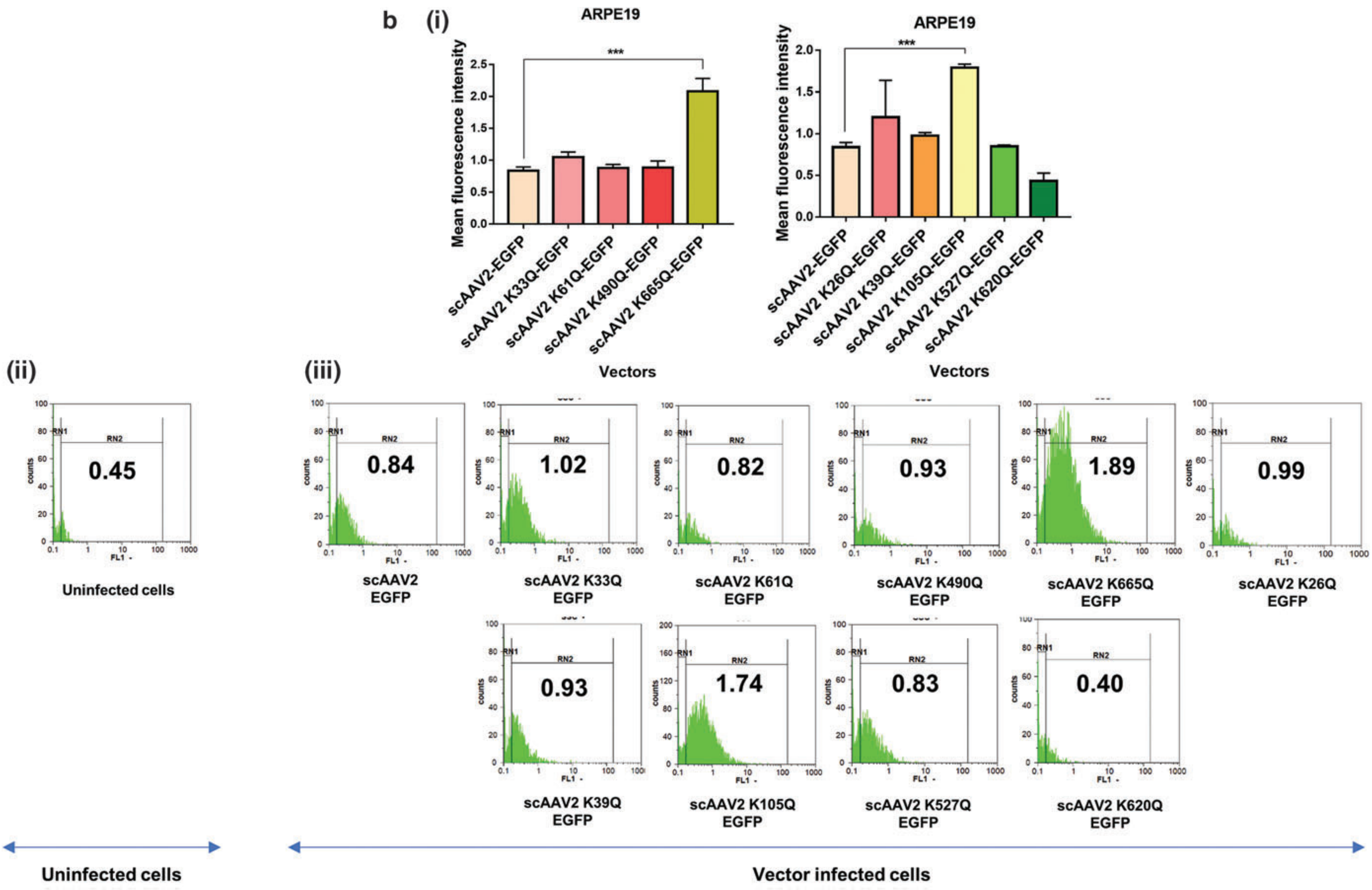

Supplementary Figure S3. (Continued).

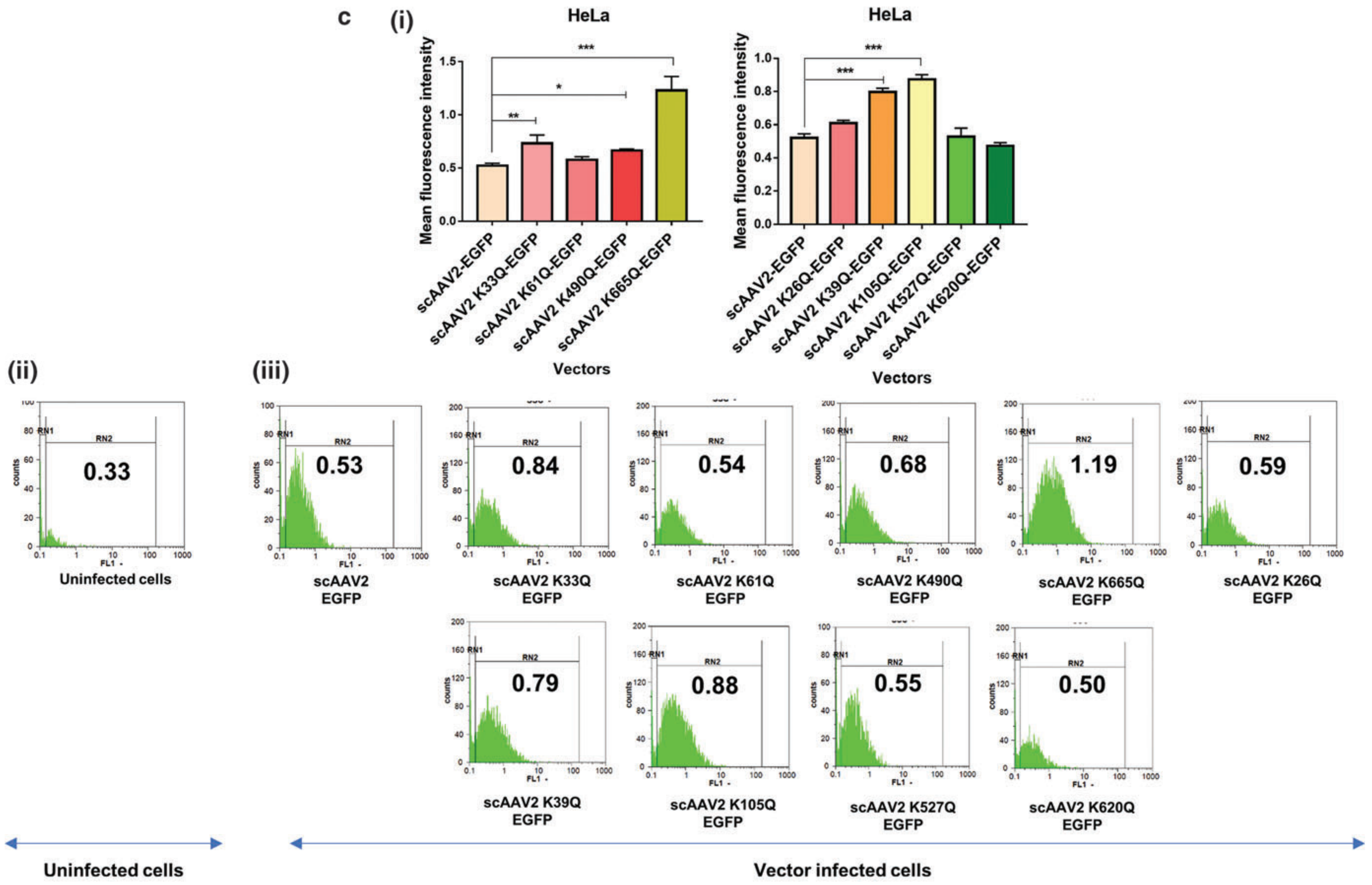

Supplementary Figure S3. (Continued).
