# Supplementary material for: Rational Engineering and Preclinical Evaluation of Neddylation and SUMOylation Site Modified Adeno-Associated Virus Vectors in Murine Models of Hemophilia B and Leber Congenital Amaurosis
Source: Hum Gene Ther. 2019 Nov 26;30(12):1461–76. doi: 10.1089/hum.2019.164 (PMC6919284; doi:10.1089/hum.2019.164)
Supplement: Supplemental data [file Supp_Fig4.pdf]

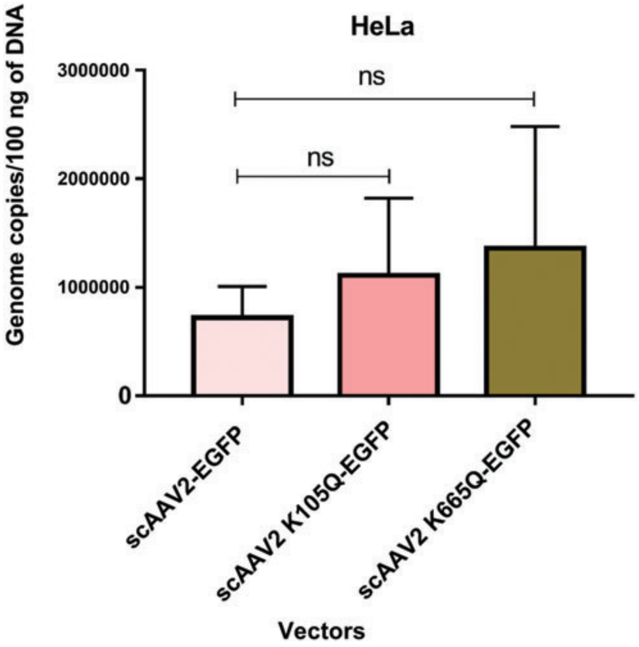

Supplementary Figure S4. Viral entry assay. HeLa cells were mock-infected or infected with scAAV2-EGFP, scAAV2 K105Q-EGFP, and scAAV2 K665Q-scEGFP mutant vector at an MOI of 1×10^4 vgs/cell. Genomic DNA was isolated after 3 h and the vector copy number was estimated by qPCR. The data represent vector copies per diploid genome and are expressed as mean $\pm$ SD from three independent experiments with three replicates for each condition. Dunnett's multiple comparisons test was used to determine the statistical significance. Data were normalized to mock condition. ns, not significant.
