# Supplementary material for: Rational Engineering and Preclinical Evaluation of Neddylation and SUMOylation Site Modified Adeno-Associated Virus Vectors in Murine Models of Hemophilia B and Leber Congenital Amaurosis
Source: Hum Gene Ther. 2019 Nov 26;30(12):1461–76. doi: 10.1089/hum.2019.164 (PMC6919284; doi:10.1089/hum.2019.164)
Supplement: Supplemental data [file Supp_Fig5.pdf]

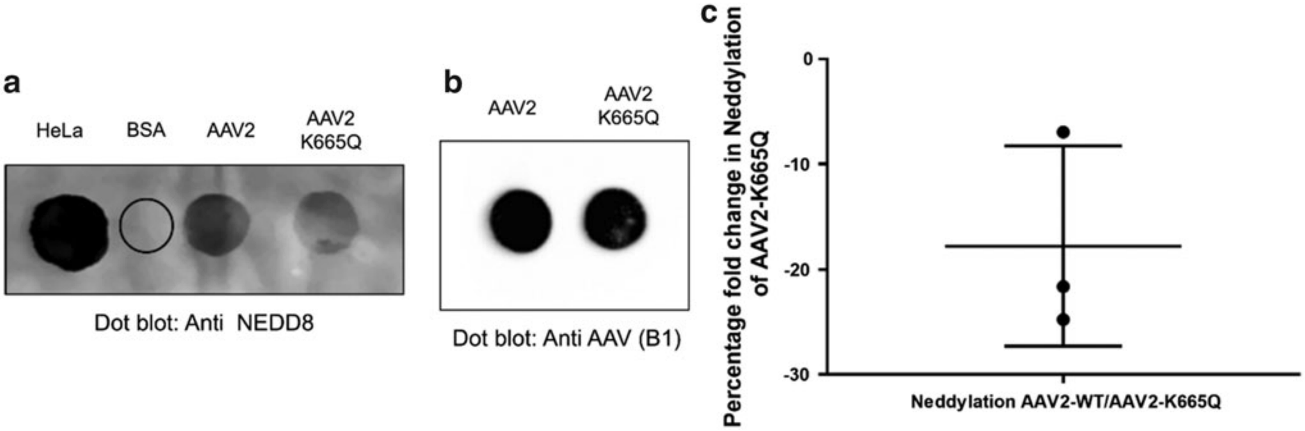

Supplementary Figure S5. Dot blot analysis for AAV2 vectors. About 1.42×10^{10} vector genomes of AAV2 and AAV2 K665Q vectors were spotted on PVDF membrane in equal volumes. The level of NEDD8 protein on vector capsids was probed by dot blot assay **(a)** and further quantified **(c)** as described in the Materials and Methods section. Anti-AAV capsid B1 antibody was used as a loading control **(b)**. The field of view pertaining to loaded samples from the entire blot is shown in the image. Exposure time for NEDD8 and B1 antibody immune-reactive blots was 9 min 47 s and 36 s, respectively, and the mean of densitometric changes between AAV2 wild-type and AAV2 K665Q vectors at these exposures is presented in **(c)**. HeLa cell lysate was used as a positive control for the assay. Data are representative from two independent biological replicates.
