# Supplementary material for: Rational Engineering and Preclinical Evaluation of Neddylation and SUMOylation Site Modified Adeno-Associated Virus Vectors in Murine Models of Hemophilia B and Leber Congenital Amaurosis
Source: Hum Gene Ther. 2019 Nov 26;30(12):1461–76. doi: 10.1089/hum.2019.164 (PMC6919284; doi:10.1089/hum.2019.164)
Supplement: Supplemental data [file Supp_Fig6.pdf]

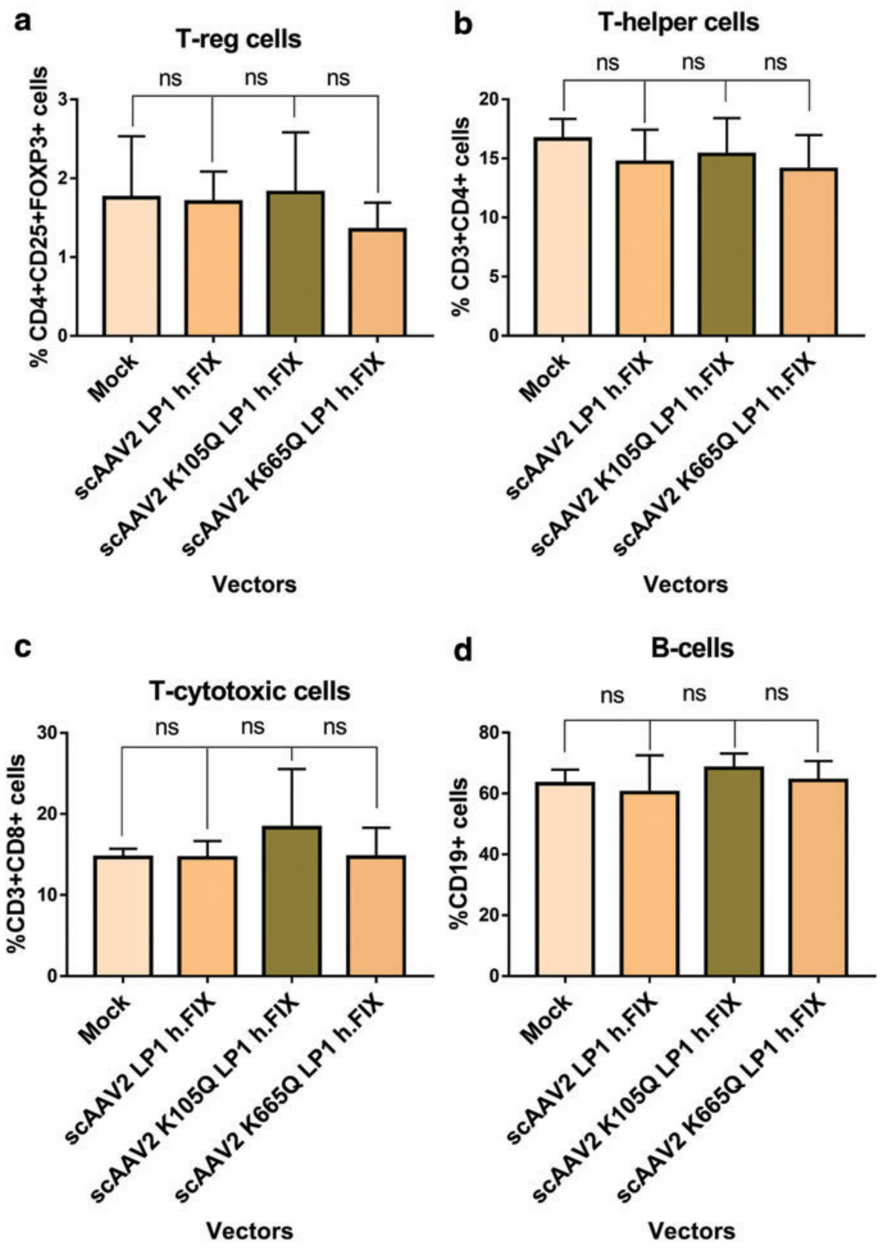

Supplementary Figure S6. Immune response in hemophilia B mice administered with AAV2 wild-type or mutant vectors. Blood samples collected at nine weeks after hepatic gene transfer were assessed for T cell and B cell markers by flow cytometry. Data for regulatory T cells ($CD4^+CD25^+$ and FoxP3) in splenocytes **(a)**, helper T cells ($CD3^+CD4^+$) **(b)**, cytotoxic T cells ($CD3^+CD8^+$ **(c)**, B lymphocytes **(d)** in peripheral blood from mock (PBS)-injected or AAV2-administered animals are shown. Dunnett's multiple comparisons test was used to determine the statistical significance. Data are expressed as mean ± SD from five animals per group. PBS, phosphate-buffered saline; ns, not significant.
