# Supplementary material for: Rational Engineering and Preclinical Evaluation of Neddylation and SUMOylation Site Modified Adeno-Associated Virus Vectors in Murine Models of Hemophilia B and Leber Congenital Amaurosis
Source: Hum Gene Ther. 2019 Nov 26;30(12):1461–76. doi: 10.1089/hum.2019.164 (PMC6919284; doi:10.1089/hum.2019.164)
Supplement: Supplemental data [file Supp_Fig7.pdf]

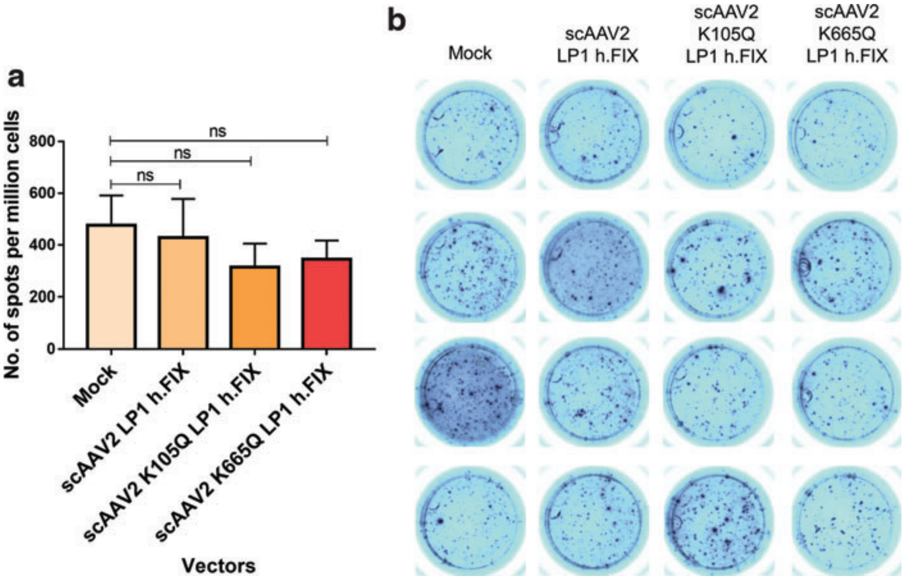

Supplementary Figure S7. IFN-γ–based ELISPOT assay to measure AAV2 capsid-specific T cell response. The graph shows the number of spots generated by 1×10^6 splenocyte cells stimulated by AAV2 capsid-specific peptide **(a)**. Representative images of generated spots from mock ($n=10$), AAV2 WT ($n=10$) and mutant treated ($n=5$) conditions are shown. Concavalin A was used as the positive control (data not shown) **(b)**. Dunnett's multiple comparisons test was used to determine the statistical significance. Data are expressed as mean $\pm$ SD of the number of spots obtained from splenocytes seeded in duplicate wells for each of the mice per group. *p*-Values were not significant ($^{ns}p>0.05$) versus mock-injected hemophilia B mice.
